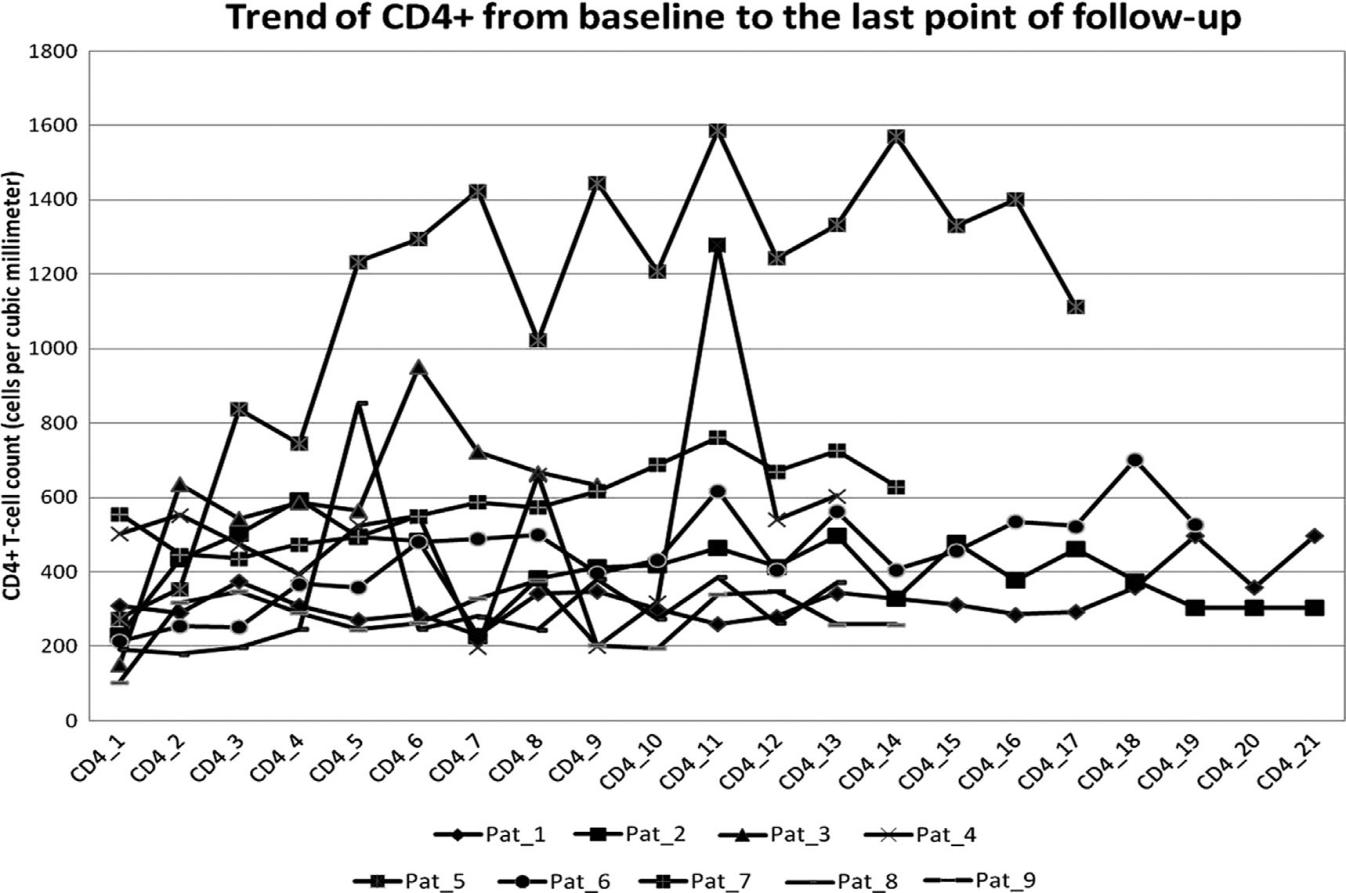# Kidney transplantation in HIV-positive patients: a report of 14 cases

**DOI:** 10.7448/IAS.15.6.18111

**Published:** 2012-11-11

**Authors:** S Casari, N Bossini, L Albini, G Setti, F Valerio, I Izzo, S Costarelli, S Sandrini, G Cancarini, F Castelli

**Affiliations:** 1 AO Spedali Civili, UO Infettivi 2, Brescia, Italy; 2 AO Spedali Civili, UO Nefrologia, Brescia, Italy

## Abstract

The HAART reduces the risk of HIV-related renal disease but the incidence of end-stage renal disease (ESRD). Therefore, efficacy and safety of renal transplantation (Tx) is an important resource in the HIV-infected population. We reported the results of kidney Tx in HIV+patients from deceased donors from June 2007 to March 2012 at our institution. The patients had to have CD4+T-cell counts≥200/mm^3^ and undetectable plasma HIV-RNA if on HAART. The induction immunosuppressive therapy consisted of metilprednisolone and basilixmab; tacrolimus and/or mycofenolic acid were used for maintenance therapy. The therapeutic drug monitoring (TDM) has been performed for the adjusting of both their doses [1]. A total of 14 patients underwent kidney Tx. They were on dialysis (haemodialysis=13, 92.9%; peritoneal=1, 7.1%) for 5±3.1 years and they were included on the Tx waiting list for 10±8 months. The baseline characteristics are showed in Table 1.

**Table:** 

**Donor at baseline**	
Mean age	38±12.5 years
Deceased	14/14 (100%)
High/unclassified infectious risk	9 (64.29%)
Recipients	
Mean age	44 years
Patients with previous AIDS-defining events	3 (21.4%)
Median follow-up months (IQR range)	42.75 (8.5–55.2)
Patient survival at last follow-up	14/14 (100%)
Graft survival at last follow-up	13/14 (92.9%)
Mean time of acute rejection since Tx	28±20 days
Patients not treated with steroid at last follow-up	6 (43%)
Plasma creatinine at last follow-up	1.87±1.93 mg/dl
Severe infectious complications (CMV pneumonia, malaria, Kaposi sarcoma)	3 (21.4%)
Diabetes	3 (21.4%)
CMV infection without localization	3 (21.4%)
Bacterial pneumonia	4 (28.6%)
Reactivation of HIV RNA	3 (21.4%)

At the last available point of follow-up (median=42.8 months, IQR=8.5–55.2), 8 out of the 13 patients (61.6%) without steroid had at least one acute rejection episode, but only 1 patient lost the graft, after 43 months (7.1%) due to chronic rejection associated with infectious and vascular complications. After Tx the median CD4+T-cell count increased from 382.5 (IQR range=233–415) to 434 (IQR range=282–605) cells/mm^3^ (p=0.055). In Figure 1 are reported the CD4+trends of 9 patients with a follow-up of at least 6 months.

HIV infection was well controlled, with only 2 (14.3%) cases of virological failure which were promptly resolved after HAART regimen modification. Table 1 shows the observed infectious complications. The skin Kaposi sarcoma has been resolved by switching to immunosuppressive therapy with sirolimus [2]. Kidney Tx appears to be safe in HIV-positive patients undergoing HAART. The viro-immunological parameters remained well controlled with no increases in infectious complications or neoplasm and a satisfactory control of HIV infection. However, the high rejection rate is a serious concern and suggests to consider a steroid-containing immunosuppressive regimen also in these patients.

**Figure:**